# Methyl iodine over oceans from the Arctic Ocean to the maritime Antarctic

**DOI:** 10.1038/srep26007

**Published:** 2016-05-17

**Authors:** Qihou Hu, Zhouqing Xie, Xinming Wang, Juan Yu, Yanli Zhang

**Affiliations:** 1 State Key Laboratory of Organic Geochemistry, Guangzhou Institute of Geochemistry, Chinese Academy of Sciences, Guangzhou, 510640, China; 2 Institute of Polar Environment, School of Earth and Space Sciences, University of Science and Technology of China, Hefei, 230026, China

## Abstract

Studies about methyl iodide (CH_3_I), an important atmospheric iodine species over oceans, had been conducted in some maritime regions, but the understanding of the spatial distribution of CH_3_I on a global scale is still limited. In this study, we reports atmospheric CH_3_I over oceans during the Chinese Arctic and Antarctic Research Expeditions. CH_3_I varied considerably with the range of 0.17 to 2.9 pptv with absent of ship emission. The concentration of CH_3_I generally decreased with increasing latitudes, except for higher levels in the middle latitudes of the Northern Hemisphere than in the low latitudes. For sea areas, the Norwegian Sea had the highest CH_3_I concentrations with a median of 0.91 pptv, while the Central Arctic Ocean had the lowest concentrations with all values below 0.5 pptv. CH_3_I concentration over oceans was affected by many parameters, including sea surface temperature, salinity, dissolved organic carbon, biogenic emissions and input from continents, with distinctive dominant factor in different regions, indicating complex biogeochemical processes of CH_3_I on a global scale.

Iodine plays an important role on atmospheric chemistry by destroying tropospheric ozone and forming new particles^1^,^2^,^3^, especially in the marine boundary layer. Among the iodine species, methyl iodide (CH_3_I) with its relatively high concentration and long lifetime (~7 days) in the atmosphere is thought to be the dominate volatile organic iodine compounds (VOICs) which works as the carrier of iodine atoms from seawater to the atmosphere^4^,^5^,^6^,^7^, although other VOICs, such as ethyl iodide (C_2_H_5_I), chloroiodomethane (CH_2_ClI), diiodomethane (CH_2_I_2_) and bromoiodomethane (CH_2_BrI)^8^,^9^,^10^,^11^, as well as inorganic iodine, such as hypoiodous acid (HOI) and I_2_^12^, are also widely detected over oceans.

CH_3_I is usually considered to be derived from oceans^13^. Emission from photochemical reactions in the surface sea water is the dominant source of CH_3_I^6^,^13^. Biogenic activity of phytoplankton and macroalgae is also its important source^14^, especially in coastal regions^15^. Besides, terrestrial ecosystems, such as rice cultivation^16^,^17^, peatland and wetland^18^, also have contribution to atmospheric CH_3_I, and are even comparable to oceanic emissions in some local areas^19^. Biomass burning releases a small quantity of CH_3_I, but its contribution is negligible on the global scale^20^. However, anthropologic activities like fossil fuel combustion and industrial emissions are not regards as the source of CH_3_I. Because the emission of CH_3_I varies with a wide range from different sources and in different regions and seasons, the estimated global flux of CH_3_I has great uncertainty^21^. More observations with comprehensive spatial and seasonal scales would help reduce the uncertainty.

There have been a considerable number of studies about CH_3_I over oceans or at coastal sites^22^,^23^,^24^,^25^,^26^,^27^,^28^,^29^,^30^. The typical concentrations of CH_3_I in the marine boundary layer were 0.1–5 pptv, with higher levels over coastal areas than remote oceans^13^. Yokouchi, *et al*.^31^ reported atmospheric CH_3_I concentrations on a wide scale including in the high, middle, and low latitudes of the both hemispheres. Recently, through several ship-based observation, Ooki, *et al*.^32^ firstly mapped CH_3_I and some other VOICs in surface seawater from the Arctic to the Antarctic, especially in the Indian Ocean, Bering Sea, and western Arctic Ocean. However, the data for the spatial distribution of CH_3_I in the marine boundary layer on a global scale is still limited, especially little study has been conducted over oceans in the high latitudes of the Northern Hemisphere like the central Arctic Ocean^33^, where abruptly sea ice change occurs due to global warming.

During the 28^th^ Chinese Antarctic Research Expedition (CHINARE 11/12, November 2011–April 2012) and the 5^rd^ Chinese Arctic Research Expedition (CHINARE 12, July–September, 2012), ambient air samples were collected in the marine boundary layer from the Arctic to the Antarctic, across more than 150° latitudes, along cruise path from the Norwegian Sea through the central Arctic Ocean, the Chukchi Sea, the western North Pacific Ocean, the eastern Indian Ocean and the southern Ocean to the maritime Antarctic. The results reveals the spatial distribution of CH_3_I over oceans on a global scale, as well as the potential sources and influencing factors of atmospheric CH_3_I, and hence provides new constraint for the model simulating the impact of CH_3_I on climate change.

## Results

### Potential CH_3_I emission from ships

CH_3_I is usually not thought to originate from anthropogenic emissions. Exceptionally, Yokouchi, *et al*.^10^ found that when air mass derived from polluted continental areas, CH_3_I mixing ratios at Hateruma Island in the East China Sea (24.05°N, 123.8°E) increased concurrently, indicating possible anthropogenic sources. However, other influencing factors, such as biogenic emissions from macroalgae in coastal seas or terrestrial ecosystems, cannot be eliminated. For samples in this study, CO was simultaneously determined with CH_3_I. In the marine boundary layer, including over coastal regions where influenced by input from continents, the concentration of CO is usually not more than 150 ppbv^34^,^35^,^36^. Extremely high CO levels indicate probable pollution by ship emissions. Although the sampling site was on the foredeck of the ship and upwind from the exhaust plume, the pollution from the ship was not absolutely excluded due to diffusive emissions^37^. As presented in Table S1 in the supplementary materials, during both the CHINARE 11/12 and the CHINARE 12, the average, maximum and median mixing ratios of CH_3_I in samples with CO concentration above 150 ppbv (CO > 150 ppbv) were much higher than those in samples with CO concentration below or equal to 150 ppbv (CO ≤ 150 ppbv). Besides, the difference between CH_3_I concentrations in samples with CO > 150 ppbv and those with CO ≤ 150 ppbv during the both two cruises was significant (heteroscedastic t-test, P < 0.05).

**Figure 1:**
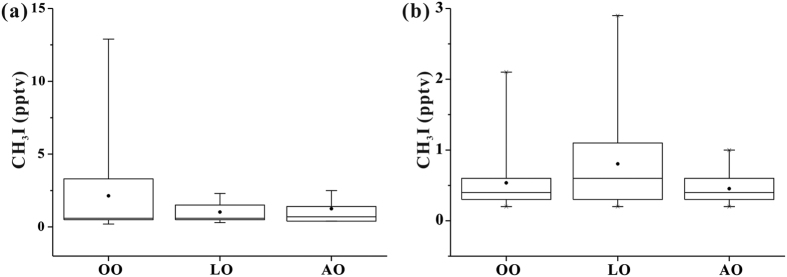
Box-and-whisker plots of CH_3_I concentrations in ocean origin (OO), land origin (LO) and Antarctic origin (AO) samples with CO concentration (**a**) below or equal to 150 ppbv and (**b**) above 150 ppbv during the CHINARE 11/12 and the CHINARE 12. The lower and upper boundaries of the box represent the 25th and the 75th percentiles, respectively; the whiskers below and above the box indicate the minimum and maximum, respectively; the line within the box marks the median; the dot represents the mean.

In order to determine the reason for the increase of CH_3_I mixing ratios in samples with CO > 150 ppbv, based on 7-day air mass back trajectories (BTs), we split these samples into three groups: ocean origin (OO), land origin (LO) and Antarctic origin (AO) as the same way of our previous study^38^. Air mass of OO samples only transported over oceans during the past 7 days, whereas air mass of AO and LO samples passed through continental Antarctica and other continents, respectively. The sampling sites when CO > 150 ppbv included both coastal regions and remote oceans (Figure S1). The mean levels of CH_3_I in LO, AO and OO samples when CO > 150 ppbv were 1.0 ± 0.69, 1.2 ± 0.93 and 2.1 ± 3.2 pptv (mean ± standard deviation, SD, the same below), respectively (Fig. 1a). There was no significant difference (heteroscedastic t-test, P > 0.05) among the three groups of samples. Emissions from macroalgae in coastal regions or terrestrial ecosystems could not explain the abruptly increased CH_3_I concentrations in OO samples when CO > 150 ppbv. Ship emission was probably caused the increase of CH_3_I concentrations in OO samples. Besides, owing to the existence of iodine in fossil fuel, such as petroleum^39^ and coal^40^, the combustion of fossil fuel may be a potential source of atmospheric CH_3_I. Direct measurement of combustion exhaust in further studies will be in favor for confirming this source.

### Spatial distribution

**Figure 2:**
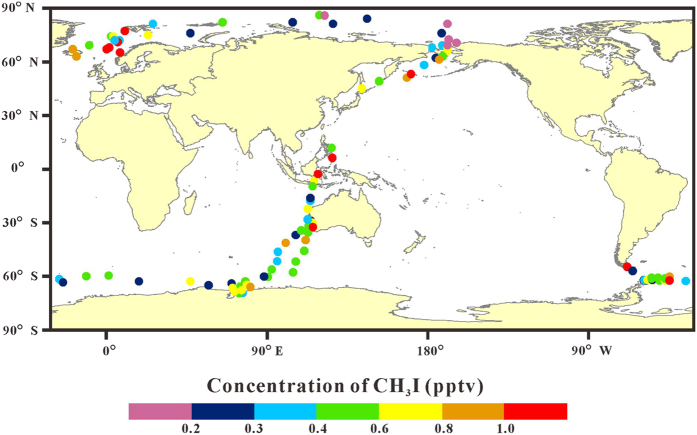
The spatial distribution of CH_3_I concentrations in the marine boundary layer when the concentration of CO below 150 pptv during the CHINARE 11/12 and the CHINARE 12. Base map is from ArcGIS 10.0 software (http://www.esri.com).

In order to avoid the disturbance from ship emission, only samples with CO ≤ 150 ppbv were selected to discuss the spatial distribution of CH_3_I concentration during the CHINARE 11/12 and CHINARE 12 (Fig. 2, Table 1). The mixing ratios of CH_3_I ranged from 0.17 to 2.9 pptv, with a mean of 0.56 ± 0.41 pptv and a median of 0.46 pptv. The mean levels of CH_3_I for LO, OO and AO samples were 0.80 ± 0.61, 0.53 ± 0.37 and 0.46 ± 0.19 pptv, respectively (Fig. 1b). CH_3_I concentrations in LO samples were significantly higher than those in OO and AO samples (P < 0.05). Emissions form macroalgaes in coastal regions can cause high CH_3_I concentrations in LO samples, while low sea surface temperature (SST) and sea ice coverage may depress CH_3_I production and sea-air exchange^5^,^41^, and thus resulted in low CH_3_I levels in AO samples. Saiz-Lopez, *et al*.^13^ summarized mixing ratios of CH_3_I in the marine boundary layer ranged as a mean level of 1.6 pptv and a median level of 1.2 pptv over coastal regions (corresponding to LO and AO samples in this study), and a mean level of 0.87 pptv and a median level of 0.70 pptv over open oceans (corresponding to OO samples in this study). It demonstrated that CH_3_I mixing ratios in the marine boundary layer in 2011–2012 were relatively lower than previous observations. It may be relevant to the SST-related decadal anomalies of CH_3_I emissions^30^. Similarly, in some coastal sites, such as Happo Ridge (36.7°N, 137.8°E), Hateruma Island (24.1°N, 123.8°E) and Cape Grim (40.4°S, 144.6°E), evident downtrend of CH_3_I concentrations were observed from 2010^30^. It should be pointed out that CH_3_I concentrations show seasonal variation, with different patterns in different latitudes^31^. In this study, due to limited observation time in each region through ship-based research, seasonal variation cannot be investigated. But the sampling time will be considered when discussing spatial distribution. Detailed results with sampling information are listed in Table S2.

CH_3_I in the marine boundary layer is principally produced through photochemical reaction in the sea surface, which is affected by solar radiation intensity and dissolved organic carbon (DOC) concentration in sea surface water^5^,^42^. The transport of CH_3_I from sea to air is controlled by SST^26^,^41^. Therefore, previous studies over the Pacific Ocean and Atlantic Ocean revealed obviously decreasing trend of CH_3_I concentrations with increasing latitudes^29^,^31^. In this study, samples in the low latitudes (30°N–30°S) were collected in the spring and autumn. According to Yokouchi, *et al*.^31^, no pronounced seasonal variation was observed in this region. CH_3_I concentration in the low latitudes ranged from 0.20 to 1.4 pptv, with a mean of 0.62 ± 0.43 pptv and a median of 0.54 pptv. The mean and median concentrations of CH_3_I in the low latitudes were higher than those in the high latitudes (60°–90°) of both hemispheres and those in the middle latitudes (30°–60°) of the Southern Hemisphere, but they were lower than those in the middle latitudes in the Northern Hemisphere (Table 1). Previous studies also indicated that the concentration of CH_3_I near the equator is slightly suppressed^31^,^41^. The chemical loss of CH_3_I in the seawater and marine boundary layer is mainly through the nucleophilic substitution reaction with chloride (Cl^−^) whose rate depends on temperature^13^,^43^. Thereby, although the production and emission of CH_3_I in the low latitudes is the rapidest, the accumulation amount of atmospheric CH_3_I may be weakened due to high loss rate. Moreover, intense convection in the tropical latitudes^44^ will accelerate the dilution of CH_3_I, and cause the decrease of CH_3_I concentration in the boundary layer^31^. Moreover, seasonal variation of CH_3_I concentration with a peak value during our sampling time in 30°–60°N is also a reason causing the lower concentrations in the low latitudes than in the middle latitudes of the Northern Hemisphere (see below).

The highest CH_3_I concentrations, with the mean of 1.1 ± 1.0 pptv and the median of 0.80 pptv, were found in the middle latitudes of the Northern Hemisphere where samples were collected in the summer. In the middle latitudes, CH_3_I concentrations reach the peak in the summer and early autumn and reach the tough in the winter^31^. High solar radiation and sea-surface DOC in the middle latitude in the summer can promote the photochemical emission of CH_3_I^13^. Besides, most samples in the middle latitudes of the Northern Hemisphere were collected in coastal regions where the emission from large algae can further enhance CH_3_I concentrations. In the western North Pacific Ocean (including the Sea of Okhotsk and the Bering Sea), CH_3_I concentrations ranged from 0.28 to 2.9 pptv, with a mean of 0.91 ± 0.83 and a median 0.74 pptv, which were comparable to the results in the early-middle autumn reported by Yokouchi, *et al*.^25^ and the results in the summer reported by Yokouchi, *et al*.^31^ Correspondingly, CH_3_I concentrations in the seawater in September–October in this region also showed high levels^32^. In the middle latitudes of the Southern Hemisphere, most samples were collected in the spring and autumn, and CH_3_I concentrations were lower than those in the middle latitudes of the Northern Hemisphere, with a mean of 0.60 ± 0.33 pptv and a median of 0.51 pptv. Over the Australian adjacent Sea, CH_3_I concentrations ranged from 0.20 to 1.3 pptv, with an average of 0.51 ± 0.31 pptv and a median of 0.51 pptv, which were near to the data reported by Yokouchi, *et al*.^31^ at Cape Grim, a coastal site of Australia (a mean of ~0.60 pptv and a median ~0.64 pptv) in the same months (March and November). Unlike previous observation over the Southern Ocean in the summer^45^, during which the peak value reached up to 2.6 pptv, CH_3_I concentrations in this study ranged from 0.24 to 0.95 pptv, with a mean of 0.51 ± 0.19 pptv and a median of 0.49 pptv. Similarly, Ooki, *et al*.^32^ reported that in the autumn, CH_3_I concentrations in the seawater in the Southern Ocean dropped compared with those in lower latitudes.

In the high latitudes of the Northern Hemisphere and the Southern Hemisphere, samples were mostly collected in the summer, and CH_3_I concentrations were in relatively low levels as a whole. The median levels in the two regions were both only 0.42 pptv. CH_3_I concentrations over the Arctic Ocean, including the Chukchi Sea and the Central Arctic Ocean, stayed in very low levels (Fig. 2), with medians of 0.22 and 0.24 pptv, respectively. Accordingly, the lowest CH_3_I concentrations in the seawater on a global scale were also found in the Chukchi Sea and the west part of the Central Arctic Ocean^32^. Especially, all the CH_3_I concentrations over the Central Arctic Ocean were below 0.5 pptv (Table 1). Low SST (about 0 °C during our sampling in the Central Arctic Ocean) may reduce the photochemical and biogenic production of CH_3_I. Moreover, sea-air exchange is also depressed by low SST and the coverage of sea ice. However, the results reported by Yokouchi, *et al*.^33^ in the Chukchi Sea and the west part of the Central Arctic Ocean in the autumn were much high than our data, with a range of 0.33–0.85 pptv and a mean of 0.52 pptv. This disparity may be caused by seasonal variation. In the high latitudes of both hemispheres, CH_3_I concentrations display a minimum in the summer and a maximum in the winter^31^. This seasonal pattern is caused by limited local emission throughout the year and more photolytic decomposition in the summer due to intense solar radiation in the high latitudes. Dramatically, over the Norwegian Sea, the average and median concentrations were as high as 0.92 ± 0.52 pptv and 0.91 pptv, respectively, which were higher than those over all the other seas. Although located in the high latitudes, the photochemical production rate and sea-air flux of CH_3_I in the Norwegian Sea are probably in high levels, because the average SST was about 10 °C during our sampling. In addition, biogenic emission may also have an important contribution owing to high oceanic primary production in the summer in the Norwegian Sea^46^. Overall, atmospheric CH_3_I concentrations in the Antarctic were relatively low, but higher than those over the Arctic Ocean. This spatial distribution pattern is also consistent with that for CH_3_I in seawater^32^. The mean and median concentrations of CH_3_I over the coastal region of the West Antarctic (including the Antarctic Peninsular and the Drake Passage) were slightly higher than those over the East Antarctic (Table 1). It may be caused by more oceanic emission in the West Antarctic due to higher SST in this region (2.3 °C in average) than in the East Antarctic (0 °C in average) during sampling.

## Discussion

### Role of sea surface temperature (SST)

The concentration of CH_3_I over oceans is mainly influenced by oceanic production, sea-air exchange and chemical loss in the seawater and atmosphere. Generally, high SST will promote the photochemical production of CH_3_I and sea-air exchange^5^,^41^, and thus correspond to high CH_3_I mixing ratios in the marine boundary layer. However, the relationship between CH_3_I concentration in the air and SST was not linear. As showed in Figure S2, atmospheric concentrations of CH_3_I increased with increasing SST and reached a peak at 10–15 °C, but rapidly deceased and stay at low levels at SST of 15–30 °C, and increased with SST above 30 °C. This trend coincides well with the pattern between SST and CH_3_I in the seawater reported by Ooki, *et al*.^32^, that CH_3_I concentrations show a peak at SST of ~15 °C and a tough at SST of ~25 °C. In the Norwegian Sea, SST ranged from 6.1 to 14 °C, and showed significantly positive correlation (R = 0.62, P < 0.05) with atmospheric CH_3_I concentration (Table 2). However, in the other regions, no obvious relationship between CH_3_I and SST was found. It may be due to complex influencing factors of atmospheric CH_3_I other than SST. For instance, in the Norwegian Sea, CH_3_I also well correlated with colored dissolved organic matter (CDOM), which is a component of DOC and can partly reflect its level (Table 2).

### Role of biogenic emission indicated by chlorophyll-a

Because CH_3_I in the marine boundary layer is affected by biogenic emission, its concentration should have relativity with chlorophyll-*a*. Previous studies reveals that CH_3_I does not present well correlation with *in-situ* chlorophyll-*a*, but significantly correlated with chlorophyll-*a* exposure along back trajectories over the past several days^45^,^47^. According to Lai, *et al*.^45^, chlorophyll-a data from NASA was taken at 6-hourly position along the back trajectories to calculate chlorophyll-a exposure over 1–7 days prior to reach the sampling site. As presented in Table 2, over the Norwegian Sea, CH_3_I concentration was significantly correlated with 1-day, 3-day and 4-day chlorophyll-*a* exposure, indicating that both local source and long-range transport of biogenic emissions affect CH_3_I in this region. CH_3_I also had significant relationship with 4-day to 7-day chlorophyll-*a* exposure in the Chukchi Sea and with 7-day chlorophyll-*a* exposure in the Australian Adjacent Sea, indicating the influence from long-range transport of biogenic emission; while significant correlation between CH_3_I and 1-day chlorophyll-*a* exposure in the Southern Ocean, indicating the contribution of local biogenic emission. In the Norwegian Sea and Southeast Asian Sea, CH_3_I also present significantly positive correlation with *α*-pinene released by phytoplankton^48^ (Table 2), indicating joint sources. For instance, *prochlorococcus* is a major biogenic source of CH_3_I^49^, and its spatial distribution, high levels in the low and middle latitudes, also agrees with that of CH_3_I. Meanwhile, *prochlorococcus* presents high *α*-pinene emission rate^48^. However, over the two seas, no significant relationship was found between CH_3_I and isoprene, another important biogenic volatile organic compound (BVOC) species over oceans^50^. This discrepancy may be due to many other functional types of phytoplankton with high isoprene emission rate, such as *synechococcus*, *haptophytes* and diatoms species^51^.

### Role of sea surface salinity and other physical factors

Significantly negative correlations between CH_3_I concentration and sea surface salinity (SSS) was found in the coastal region of the West Antarctic (R = −0.57, P < 0.05). Similarly, the decreasing trend of CH_3_I concentration in the seawater with the increasing SSS was observed at Kiel Fjord in the Baltic Sea^52^. High SSS value means high Cl^−^ in the seawater, and thus causes more CH_3_I depleted in the seawater. However, the parameters link to production and sea-air exchange of CH_3_I, such as SST, wind speed (WD) and CDOM did not present obvious relationship with atmospheric CH_3_I in the West Antarctic. It indicated that rather than emission, chemical loss dominated CH_3_I concentrations in this region. Besides these factor, in the western North Pacific Ocean, dramatically, CH_3_I showed significant correlation with CO (R = 0.73, P < 0.05), indicating combustion emissions. CH_3_I concentrations in LO samples (0.80–2.9 pptv) were much higher than those in OO samples (0.31–0.68 pptv), further suggesting the input from continents. This region is heavily affected by biomass burning in the East Siberia^53^, which can also emit CH_3_I^54^. Besides, anthropogenic sources like fossil fuel burning possibly also played a role on CH_3_I over oceans^10^. In the Central Arctic Ocean, the Barents Sea and the coastal region of the East Antarctic Ocean, no parameter was found to be significantly correlated with CH_3_I concentration. It may be caused by the offset of the role of each factor and too low CH_3_I levels in these seas.

## Experimental Methods

### Sampling

Ambient air samples were collected between the East China Sea to the coastal regions of Antarctica (35°N–70°S) during the CHINARE 11/12 and between the East China Sea to the Arctic Ocean (37°N–88°N) during the CHINARE 12. The sampling site located upwind on the upper-most deck of the icebreaker *Xuelong*. 2-L electro-polished stainless steel canisters, which can keep gases in it out of light and avoid photochemical reaction, were used to collect ambient air samples. Prior to the cruises, the canisters were cleaned and evacuated. Each sampling lasted for about 5 minutes. After sampling, the canisters were then stored in a dark and thermostatic room at 4 °C during the cruises. After the cruises, samples were sent to the Guangzhou Institute of Geochemistry (GIG), Chinese Academy of Sciences, for analysis immediately. All the analysis was done within 6 months after collection for the CHINARE 11/12 samples, and within 3 months for the CHINARE 12 samples. Yokouchi, *et al*.^50^ reported that CH_3_I does not show significant decline in canisters 6-month after sampling.

### Chemical analysis

A Model 7100 pre-concentrator (Entech Instruments Inc., California, USA) coupled with an Agilent 5973 N gas chromatography-mass selective detector/flame ionization detector (GC-MSD/FID, Agilent Technologies, USA) was used to analyse volatile organic compounds (VOCs) in the canister samples. Details of the analytical procedure were previously described^55^. Briefly, 500 mL air sample in the canister was first concentrated in a liquid-nitrogen cryogenic trap at −160 °C. Then, pure helium transferred the trapped VOCs to a secondary trap at −40 °C with Tenax-TA as an adsorbent. During these two processes, the majority of H_2_O and CO_2_ were removed. The secondary trap was then heated to transfer the target VOCs to a third cryo-focus trap at −170 °C by helium. The third trap was heated rapidly to transfer the VOCs into the GC-MSD/FID system. Helium was used with a HP-1 capillary column (60 m × 0.32 mm × 1.0 μm, Agilent Technologies, USA) as carrier gas and then divided in two ways: The first was a PLOT-Q column (30 m × 0.32 mm × 2.0 μm, Agilent Technologies, USA) followed by FID detection. The second was a 65 cm × 0.10 mm I.D stainless steel line followed by MSD detection. The GC oven temperature was initially set at −50 °C for 3 min and increased to 10 °C at 15 °C min^−1^, then 120 °C at 5 °C min^−1^, then 250 °C at 10 °C min^−1^ and remaining at 250 °C for 10 min. The MSD was used in selected ion monitoring (SIM) mode and the ionization method was electron impact ionization (EI). CH_3_I concentrations were obtained from the signal of MSD. CO in the canister was separated by a packed column (5 Å Molecular Sieve 60/80 mesh, 3 m × 1/8 in.), converted to CH_4_ by a Ni-based catalyst, and then analyzed by an Agilent 6890 gas chromatograph equipped with an FID.

### Quality Control and Assurance

Before the cruises, all canisters were cleaned at least five times by filling and evacuating with humidified zero air. In order to check any possible contamination in the canisters, all canisters were evacuated after the cleansing procedures, re-filled with pure nitrogen, stored in the laboratory for at least 24 h, and then the same methods as field samples were used to analyse VOCs and ensure that no target compounds were found or that they were under the method detection limit. CH_3_I was identified based on its retention times and mass spectra, and quantified by a mixture standard with 0.58 pptv of CH_3_I in it from the Rowland/Blake laboratory at the University of California, Irvine^56^. The comparison of quantification between the GIG laboratory and the Rowland/Blake laboratory had been conducted through duplicate samples^57^. The relative measurement deviations were within 4% for CH_3_I. Before sample analysis, the analytical system was checked daily with a one-point calibration. If the results were beyond ±10% of the initial calibration curve, recalibration was performed. The detection limit for CH_3_I in this study is ~0.10 pptv.

### Data of chlorophyll-*a*, sea ice and air mass back trajectories

Satellite chlorophyll-a data in the surface seawater were obtained by Moderate Resolution Imaging Spectroradiometer (MODIS) from NASA satellites (http://oceancolor.gsfc.nasa.gov). Air mass back trajectories (BTs) were calculated for the samples using HYSPLIT (HYbrid Single-Particle Lagrangian Integrated Trajectory) transport and dispersion model from the NOAA Air Resources Laboratory (http://www.arl.noaa.gov/ready/hysplit4.html). 7-day BTs for each sampling were traced with 6 h steps at 50 m above sea level.

## Additional Information

**How to cite this article**: Hu, Q. *et al*. Methyl iodine over oceans from the Arctic Ocean to the maritime Antarctic. *Sci. Rep.* **6**, 26007; doi: 10.1038/srep26007 (2016).

## Supplementary Material

Supplementary Information

## Figures and Tables

**Table 1:** Range, mean (±SD) and median of CH_3_I (pptv) in different regions during the CHINARE 11/12 and the CHINARE 12.

Region	range	Mean ( ± SD)	median
East Antarctic	0.20–0.89	0.46 ± 0.21	0.37
West Antarctic	0.22–1.6	0.56 ± 0.38	0.42
Southern Ocean	0.24–0.95	0.51 ± 0.19	0.49
Australian adjacent Sea	0.20–1.3	0.51 ± 0.31	0.51
Southeast Asia Sea	0.49–1.4	0.90 ± 0.45	0.62
west North pacific	0.28–2.9	0.91 ± 0.83	0.74
Chukchi Sea	0.17–0.70	0.30 ± 0.19	0.22
Central Arctic Ocean	0.17–0.46	0.28 ± 0.12	0.24
Barents Sea	0.22–0.67	0.43 ± 0.23	0.39
Norwegian Sea	0.30–2.1	0.92 ± 0.52	0.91
60°S–90°S	0.20–1.6	0.48 ± 0.25	0.42
30°S–60°S	0.24–1.5	0.60 ± 0.33	0.51
30°N–30°S	0.20–1.4	0.62 ± 0.43	0.54
30°N–60°N	0.31–2.9	1.1 ± 1.0	0.80
60°N–90°N	0.17–2.1	0.56 ± 0.44	0.42
Southern Hemisphere	0.20–1.6	0.52 ± 0.29	0.46
Northern Hemisphere	0.17–2.9	0.65 ± 0.56	0.47

**Table 2:** Correlation coefficients of CH_3_I with the sea surface temperature (SST), wind speed (WD), sea surface salinity (SSS), colored dissolved organic matter (CDOM), concentrations of isoprene, α-pinene and carbon monoxide (CO), and 1-day to 7-day chlorophyll-*a* exposure in different regions during the CHINARE 11/12 and the CHINARE 12*.

Region	SST	WD	SSS	CDOM	isoprene	α-pinene	CO	chlorophyll-*a* exposure						
								1-day	2-day	3-day	4-day	5-day	6-day	7-day
East Antarctic	−0.39	0.28	0.06	−0.30	−0.26	−0.23	0.05	−0.12	−0.02	−0.16	−0.07	−0.10	−0.18	−0.19
West Antarctic	0.28	−0.15	**−0.57**	−0.40	−0.09	−0.19	−0.14	−0.22	0.03	0.00	−0.07	0.01	−0.11	−0.13
Southern Ocean	0.21	−0.49	0.24	−0.18	−0.02	0.22	0.03	**0.65**	0.41	0.36	0.41	−0.28	−0.26	−0.26
Australian adjacent Sea	−0.08	0.45	0.51	−0.52	−0.16	−0.27	0.09	−0.05	−0.04	0.07	−0.02	0.07	0.49	**0.67**
Southeast Asia Sea	0.45	−0.50	−0.55	−0.40	−0.83	**0.89**	−0.09	0.51	0.45	0.44	0.38	0.25	0.03	−0.14
West North pacific	−0.16	−0.04	0.40	0.04	0.30	0.42	**0.73**	0.26	0.02	−0.01	0.01	0.01	0.01	0.04
Chukchi Sea	−0.02	−0.04	−0.27	0.72	−0.23	_^a^	0.38	0.72	0.80	0.79	**0.98**	**0.99**	**0.98**	**0.96**
Central Arctic Ocean	0.28	−0.15	−0.57	−0.40	−0.09	−0.19	−0.14	−0.22	0.03	0.00	−0.07	0.01	−0.11	−0.13
Barents Sea	0.24	−0.92	0.17	−0.39	0.93	0.93	−0.14	_^b^	_^b^	_^b^	_^b^	_^b^	_^b^	_^b^
Norwegian Sea	**0.62**	0.53	−0.18	**0.71**	0.19	**0.75**	0.50	**0.65**	0.19	**0.66**	**0.70**	0.30	0.13	0.09

^*^Values with P < 0.05 are in bold.

^a^α-pinene was not detected in the Chukchi Sea.

^b^Satellite chlorophyll-a data was lack in the Barents Sea during sampling.
