# Supplementary material for: Methyl iodine over oceans from the Arctic Ocean to the maritime Antarctic
Source: Sci Rep. 2016 May 17;6:26007. doi: 10.1038/srep26007 (PMC4868973; doi:10.1038/srep26007)
Supplement: Supplementary Information [file srep26007-s1.pdf]

Supplementary information for the manuscript:

Methyl iodine over oceans from the Arctic Ocean to the maritime Antarctic

Qihou Hu[1,2], Zhouqing Xie[2*], Xinming Wang[1], Juan Yu[2], Yanli Zhang[1]

1. State Key Laboratory of Organic Geochemistry, Guangzhou Institute of Geochemistry, Chinese Academy of Sciences, Guangzhou, 510640, China
2. Institute of Polar Environment, School of Earth and Space Sciences, University of Science and Technology of China, Hefei, 230026, China

*Correspondence and requests for materials should be addressed to Z.Q.X. (zqxie@ustc.edu.cn)

To complement the information given in the main manuscript, the following sections provide supporting information: Figures S1 and S2, and Tables S1and S2.

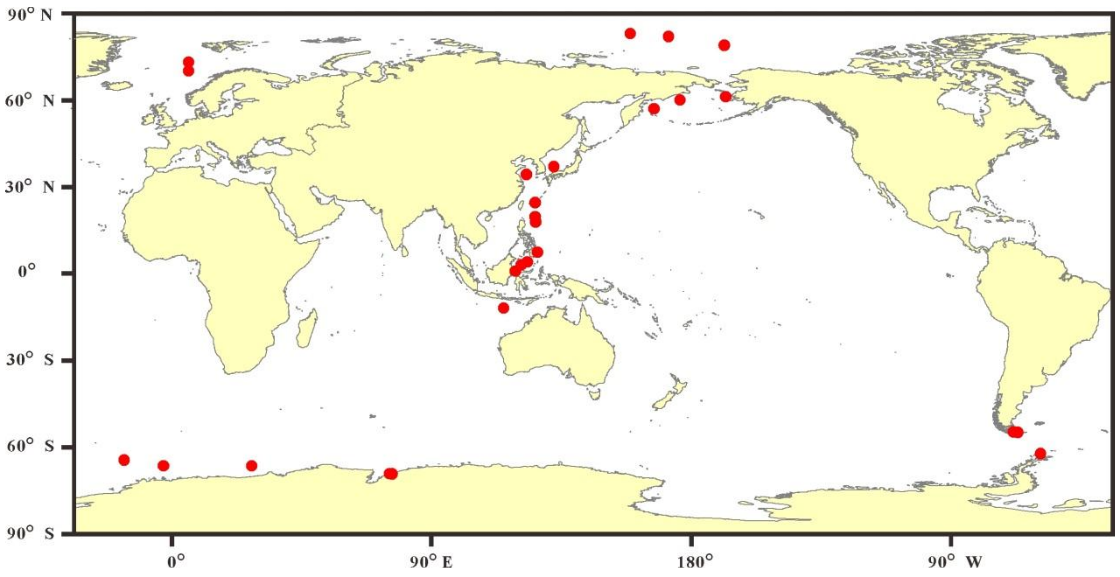

Figure S1 Sampling sites when CO>150 pptv during the CHINARE 11/12 and the CHINARE 12. Base map is from ArcGIS 10.0 software (http://www.esri.com).

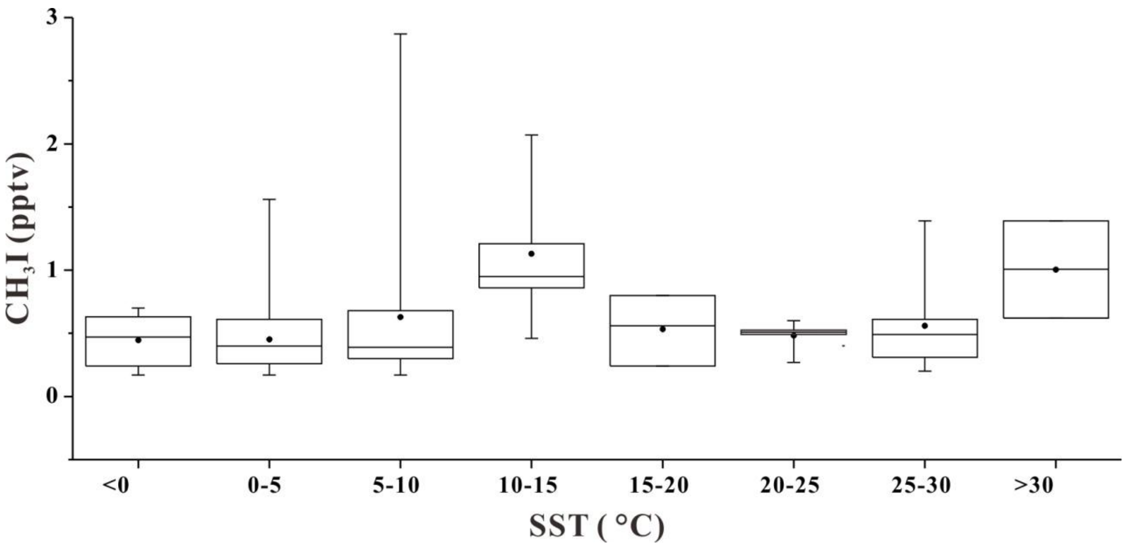

Figure S2 Box-and-whisker plots of atmospheric CH_3I concentrations with different sea surface temperature (SST) ranges during the CHINARE 11/12 and the CHINARE 12. The lower and upper boundaries of the box represent the 25th and the 75th percentiles, respectively; the whiskers below and above the box indicate the minimum and maximum, respectively; the line within the box marks the median; the dot represents the mean.

Table S1. Comparison of CH_3I concentrations (pptv) with CO concentration below or equal to 150 ppbv and those with CO concentration above 150 ppbv during the CHINARE 11/12 and the CHINARE 12.

Cruise	CH_3I (CO ≤ 150 ppbv)			CH_3I (CO > 150 ppbv)			P[*]
	Range	Mean	Median	Range	Mean	median	
CHINARE 11/12	0.20-1.6	0.53	0.47	0.29-3.4	1.2	0.65	<0.01
CHINARE 12	0.17-2.9	0.63	0.44	0.23-12.9	2.6	1.5	<0.01

[*] P value of difference examination (heteroscedastic t-test) between samples with CO ≤ 150 ppbv and those with CO > 150 ppbv.

Table S2. Sampling information, concentrations of CH_3I and CO[*], and classification based on 7-day air mass back trajectories[**].

Sampling date	Latitude[***]	Longitude[***]	CH_3I (pptv)	CO (ppbv)	Air mass origin
**Oct. 30, 2011**	**34.25**	**122.94**	**0.4**	**404**	**LO**
**Nov. 6, 2011**	**24.59**	**126.00**	**0.6**	**205**	**OO**
**Nov. 7, 2011**	**19.58**	**126.00**	**0.6**	**153**	**OO**
**Nov. 9, 2011**	**7.39**	**126.77**	**0.3**	**153**	**LO**
**Nov. 11, 2011**	**0.75**	**119.14**	**1.7**	**594**	**LO**
Nov. 12, 2011	-6.50	116.61	0.62	135	OO
**Nov. 13, 2011**	**-12.01**	**115.19**	**2.3**	**509**	**LO**
Nov. 14, 2011	-17.97	114.24	0.32	115	LO
Nov. 16, 2011	-29.01	114.39	0.27	89	LO
Nov. 19, 2011	-32.05	115.74	1.25	94	LO
Nov. 20, 2011	-35.56	112.83	0.56	84	OO
Nov. 21, 2011	-40.03	111.63	0.89	64	OO
Nov. 22, 2011	-45.80	110.78	0.46	139	OO
Nov. 23, 2011	-51.97	106.12	0.56	83	OO
Nov. 24, 2011	-57.85	104.54	0.41	71	OO
Nov. 26, 2011	-60.50	90.61	0.52	72	OO
Nov. 27, 2011	-62.91	77.87	0.45	72	OO
Nov. 28, 2011	-65.88	77.35	0.89	73	OO
Nov. 29, 2011	-69.28	76.37	0.28	66	AO
Nov. 30, 2011	-69.28	76.37	0.37	72	AO
Dec. 6, 2011	-69.29	76.38	0.85	77	OO
Dec. 8, 2011	-69.28	76.37	0.20	82	AO
Dec. 12, 2011	-69.28	76.37	0.28	61	AO
Dec. 18, 2011	-65.81	74.96	0.47	59	AO

Dec. 19, 2011	-64.21	70.24	0.24	101	AO
Dec. 21, 2011	-63.00	47.18	0.70	123	OO
Dec. 23, 2011	-63.00	18.41	0.22	65	OO
Dec. 25, 2011	-59.79	1.37	0.49	72	OO
Dec. 26, 2011	-60.06	-11.23	0.60	67	OO
Dec. 28, 2011	-61.63	-26.19	0.30	67	OO
Dec. 31, 2011	-62.02	-53.33	0.70	74	AO
Jan. 3, 2012	-62.23	-58.93	0.22	65	OO
**Jan. 4, 2012**	**-62.23**	**-58.93**	**3.3**	**363**	**OO**
Jan. 5, 2012	-62.36	-58.27	0.23	62	AO
**Jan. 6, 2012**	**-62.23**	**-58.91**	**1.4**	**306**	**OO**
Jan. 7, 2012	-62.32	-58.66	0.37	80	OO
**Jan. 9, 2012**	**-54.95**	**-66.96**	**0.6**	**178**	**LO**
Jan. 12, 2012	-54.81	-68.27	1.46	65	LO
**Jan. 14, 2012**	**-54.81**	**-68.26**	**0.8**	**169**	**LO**
Jan. 15, 2012	-57.18	-65.30	0.27	72	OO
Jan. 17, 2012	-62.09	-56.86	0.62	70	OO
Jan. 19, 2012	-62.20	-53.67	0.61	69	OO
Jan. 20, 2012	-62.04	-54.31	0.26	70	OO
Jan. 21, 2012	-61.35	-54.61	0.41	105	OO
Jan. 23, 2012	-60.81	-54.62	0.40	59	OO
Jan. 24, 2012	-60.86	-50.99	0.43	48	AO
Jan. 25, 2012	-62.52	-50.28	0.42	61	AO
Jan. 26, 2012	-62.39	-47.15	0.69	80	AO
Jan. 27, 2012	-61.29	-47.17	0.51	59	OO
Jan. 28, 2012	-60.35	-44.67	0.86	59	OO
Jan. 29, 2012	-62.39	-44.69	1.56	57	OO
Jan. 30, 2012	-62.85	-35.60	0.38	61	OO
Jan. 31, 2012	-63.57	-24.13	0.29	61	OO
**Feb. 1, 2012**	**-64.40**	**-16.37**	**0.4**	**195**	**OO**
**Feb. 2, 2012**	**-66.58**	**-2.82**	**3.4**	**169**	**OO**
**Feb. 4, 2012**	**-66.58**	**27.80**	**0.5**	**271**	**OO**
Feb. 6, 2012	-65.01	57.28	0.24	60	AO
Feb. 7, 2012	-66.70	70.77	0.65	60	AO
Feb. 8, 2012	-69.11	75.93	0.37	75	AO
Feb. 10, 2012	-69.07	75.72	0.25	49	AO
Feb. 12, 2012	-69.35	76.45	0.67	57	AO
Feb. 13, 2012	-69.33	76.49	0.25	128	AO
**Feb. 15, 2012**	**-69.33**	**76.44**	**1.4**	**207**	**AO**
Feb. 16, 2012	-69.23	75.34	0.27	51	AO
Feb. 18, 2012	-69.32	76.46	0.36	68	AO
**Feb. 20, 2012**	**-69.36**	**76.46**	**0.4**	**205**	**AO**
**Feb. 21, 2012**	**-69.33**	**76.48**	**0.7**	**264**	**AO**
Feb. 24, 2012	-69.21	75.87	0.37	68	AO

Feb. 26, 2012	-69.47	74.41	0.63	55	AO
Feb. 27, 2012	-69.47	74.41	0.57	110	AO
Feb. 28, 2012	-69.46	74.46	0.54	59	AO
Feb. 29, 2012	-68.48	71.94	0.64	95	AO
Mar. 2, 2012	-67.97	76.02	0.70	48	AO
**Mar. 4, 2012**	**-69.20**	**75.62**	**2.5**	**159**	**AO**
Mar. 7, 2012	-64.95	78.57	0.62	61	AO
Mar. 8, 2012	-60.30	88.32	0.21	53	OO
Mar. 9, 2012	-56.24	92.73	0.46	55	AO
Mar. 10, 2012	-51.80	95.85	0.35	63	AO
Mar. 11, 2012	-46.47	96.17	0.39	77	OO
Mar. 12, 2012	-41.40	100.49	0.95	72	AO
Mar. 13, 2012	-37.12	106.12	0.24	55	OO
Mar. 14, 2012	-34.49	109.04	0.51	57	OO
Mar. 16, 2012	-31.90	115.65	0.51	78	LO
Mar. 17, 2012	-31.90	115.65	0.60	149	LO
Mar. 19, 2012	-31.90	115.65	0.52	96	LO
Mar. 25, 2012	-28.39	112.81	0.31	103	LO
Mar. 26, 2012	-22.43	112.99	0.61	79	LO
Mar. 27, 2012	-16.32	114.23	0.20	73	OO
Mar. 28, 2012	-9.92	115.48	0.60	69	OO
Mar. 29, 2012	-2.91	118.56	1.39	79	LO
**Mar. 30, 2012**	**2.94**	**121.25**	**0.4**	**178**	**OO**
**Mar. 31, 2012**	**3.98**	**123.33**	**1.6**	**159**	**OO**
Arp. 1, 2012	6.16	126.64	1.39	132	OO
Arp. 2, 2012	11.79	126.17	0.49	126	LO
**Arp. 3, 2012**	**17.89**	**126.12**	**0.6**	**151**	**OO**
**Jul. 4, 2012**	**37.01**	**132.54**	**1.5**	**163**	**LO**
Jul. 6, 2012	45.01	143.16	0.80	136	LO
Jul. 8, 2012	49.01	152.66	0.49	108	OO
Jul. 10, 2012	51.01	168.44	0.98	105	LO
Jul. 11, 2012	53.01	170.72	2.87	145	LO
Jul. 13, 2012	58.01	177.84	0.31	105	OO
Jul. 16, 2012	62.00	-175.42	0.28	96	OO
Jul. 18, 2012	66.01	-168.92	0.68	109	OO
Jul. 19, 2012	69.00	-168.87	0.18	100	OO
Jul. 20, 2012	70.01	-163.41	0.17	84	LO
Jul. 21, 2012	69.00	-171.88	0.36	105	OO
Jul. 23, 2012	68.00	-177.59	0.31	128	LO
Aug. 1, 2012	76.00	47.25	0.22	91	OO
Aug. 2, 2012	75.00	23.53	0.67	91	OO
Aug. 3, 2012	74.01	2.75	0.47	86	OO
**Aug. 4, 2012**	**70.01**	**6.04**	**4.0**	**185**	**OO**
Aug. 5, 2012	72.01	7.38	0.30	98	OO

Aug. 7, 2012	74.00	3.89	0.61	94	OO
**Aug. 8, 2012**	**73.01**	**6.06**	**12.9**	**627**	**OO**
Aug. 9, 2012	71.00	6.43	1.29	116	OO
Aug. 10, 2012	68.01	1.74	1.14	126	LO
Aug. 11, 2012	67.01	0.23	2.07	129	OO
Aug. 12, 2012	65.01	7.73	1.27	122	OO
Aug. 13, 2012	63.00	-16.65	0.86	102	LO
Aug. 21, 2012	67.00	-18.82	0.96	119	OO
Aug. 22, 2012	69.01	-9.53	0.51	123	OO
Aug. 23, 2012	72.01	4.61	0.33	129	LO
Aug. 24, 2012	77.00	10.30	1.21	149	LO
Aug. 25, 2012	81.00	26.16	0.39	128	OO
Aug. 26, 2012	82.00	65.13	0.42	119	OO
Aug. 27, 2012	82.00	104.32	0.21	100	OO
Aug. 28, 2012	81.01	127.02	0.24	118	OO
Aug. 29, 2012	86.01	120.79	0.17	134	OO
Aug. 31, 2012	86.00	119.49	0.46	124	OO
Sep. 1, 2012	84.01	145.98	0.25	126	OO
**Sep. 2, 2012**	**83.01**	**159.04**	**1.7**	**296**	**OO**
**Sep. 3, 2012**	**82.01**	**172.33**	**0.5**	**155**	**OO**
Sep. 4, 2012	81.01	-168.91	0.20	129	OO
**Sep. 5, 2012**	**79.00**	**-168.46**	**0.2**	**154**	**OO**
Sep. 6, 2012	76.00	-172.15	0.22	128	OO
Sep. 7, 2012	72.01	-168.90	0.17	132	OO
Sep. 8, 2012	68.01	-168.90	0.70	130	OO
Sep. 9, 2012	63.01	-171.69	0.42	150	LO
**Sep. 10, 2012**	**61.01**	**-168.01**	**0.5**	**172**	**LO**
Sep. 11, 2012	61.00	-173.43	0.91	136	LO
**Sep. 12, 2012**	**60.00**	**176.21**	**1.5**	**160**	**LO**
**Sep. 13, 2012**	**57.01**	**167.16**	**0.6**	**171**	**LO**

* Samples with CO>150 ppbv are in bold.

** Based on 7-day air mass back trajectories (BTs), samples were divided into three groups: ocean origin (OO), land origin (LO) and Antarctic origin (AO) ocean origin (OO), land origin (LO) and Antarctic origin (AO). Air mass of OO samples only transported over oceans during the past 7 days, whereas air mass of AO and LO samples passed through continental Antarctica and other continents, respectively.

*** The latitude and longitude represent the mean location of the start and end of each sampling episode. Sample sites in latitude south and north are denoted with "-" and "+", respectively. Sample sites in longitude west and east are denoted with "-" and "+", respectively.
